# Interventions to promote cost-effectiveness in adult intensive care units: consensus statement and considerations for best practice from a multidisciplinary and multinational eDelphi study

**DOI:** 10.1186/s13054-023-04766-2

**Published:** 2023-12-11

**Authors:** Amit Kansal, Jos M. Latour, Kay Choong See, Sumeet Rai, Maurizio Cecconi, Carl Britto, Andrew Conway Morris, Raymond Dominic Savio, Vinay M. Nadkarni, B. K. Rao, Rajesh Mishra

**Affiliations:** 1 Department of Intensive Care Medicine, Ng Teng Fong General Hospital, Jurong Health Campus, National University Health System, Singapore, Singapore; 2 School of Nursing and Midwifery, Faculty of Health, University of Plymouth, Plymouth, UK; 3 Division of Respiratory and Critical Care Medicine, Department of Medicine, National University Hospital, Singapore, Singapore; 4 Intensive Care Unit, Canberra Hospital, Canberra, Australia; 5 Department of Biomedical Sciences, Humanitas University, Via Rita Levi Montalcini 4, 20072 Pieve Emanuele, Milan, Italy; 6 Division of Critical Care, Department of Anesthesia, Critical Care and Pain Medicine, Boston Children’s Hospital, Boston, USA; 7 Division of Anaesthesia, Department of Medicine, University of Cambridge, Cambridge, UK; 8 Critical Care Services, Apollo Proton Cancer Center, Chennai, India; 9 Department of Anesthesiology, Critical Care, and Pediatrics at the Children’s Hospital of Philadelphia (CHOP), University of Pennsylvania Perelman School of Medicine, Philadelphia, USA; 10 Department of Critical Care Medicine, Sir Ganga Ram Hospital, New Delhi, India; 11 Shaibya Comprehensive Care Clinic, Ahmedabad, India; 12 IRCCS Humanitas Research Hospital, Via Manzoni 56, 20089 Rozzano, Milan, Italy; 13 Department of Nursing, Zhongshan Hospital, Fudan University, Shanghai, China; 14 John V Farman Intensive Care Unit, Addenbrooke’s Hospital, Cambridge University Hospitals NHS Foundation Trust, Cambridge, UK

**Keywords:** Delphi technique, Healthcare costs, Health resources, Intensive care units, Quality of health care

## Abstract

**Background:**

There is limited evidence to guide interventions that promote cost-effectiveness in adult intensive care units (ICU). The aim of this consensus statement is to identify globally applicable interventions for best ICU practice and provide guidance for judicious use of resources.

**Methods:**

A three-round modified online Delphi process, using a web-based platform, sought consensus from 61 multidisciplinary ICU experts (physicians, nurses, allied health, administrators) from 21 countries. Round 1 was qualitative to ascertain opinions on cost-effectiveness criteria based on four key domains of high-value healthcare (foundational elements; infrastructure fundamentals; care delivery priorities; reliability and feedback). Round 2 was qualitative and quantitative, while round 3 was quantitative to reiterate and establish criteria. Both rounds 2 and 3 utilized a five-point Likert scale for voting. Consensus was considered when > 70% of the experts voted for a proposed intervention. Thereafter, the steering committee endorsed interventions that were identified as ‘critical’ by more than 50% of steering committee members. These interventions and experts’ comments were summarized as final considerations for best practice.

**Results:**

At the conclusion of round 3, consensus was obtained on 50 best practice considerations for cost-effectiveness in adult ICU. Finally, the steering committee endorsed 9 ‘critical’ best practice considerations. This included adoption of a multidisciplinary ICU model of care, focus on staff training and competency assessment, ongoing quality audits, thus ensuring high quality of critical care services whether within or outside the four walls of ICUs, implementation of a dynamic staff roster, multidisciplinary approach to implementing end-of-life care, early mobilization and promoting international consensus efforts on the Green ICU concept.

**Conclusions:**

This Delphi study with international experts resulted in 9 consensus statements and best practice considerations promoting cost-effectiveness in adult ICUs. Stakeholders (government bodies, professional societies) must lead the efforts to identify locally applicable specifics while working within these best practice considerations with the available resources.

**Supplementary Information:**

The online version contains supplementary material available at 10.1186/s13054-023-04766-2.

## Background

There is a growing recognition among healthcare stakeholders of the responsibility of providers to deliver value-based healthcare with the costs of care assessed alongside outcome [1, 2]. Intensive care treatments represent a significant proportion of increasing healthcare costs [3]. To comprehensively assess the economic viability of various interventions, cost-effectiveness analysis serves as a valuable tool, examining both the costs and health outcomes associated with these interventions [4]. Despite the high costs involved, there are very few cost-effectiveness studies in intensive care units (ICUs), with an average of 4.6 studies published per year [4].

Numerous policies and research efforts have aimed to improve the quality of critical care delivery, however less attention has been focused on cost-effectiveness [3]. Only a few interventions have demonstrated improved clinical outcomes in critical care. Heterogeneity among critical care patients who present with varied diagnoses and require a range of different interventions also make it difficult to conduct cost-effectiveness studies focused on single items. In addition, there are no universally acceptable ways to measure ICU-related costs [5–12]. Most studies describe projected benefits or estimate potential cost savings only [5]. Our study aimed to go beyond cost-containment and sought to identify measures which could maximize care quality and outcomes within existing funding envelopes. Previous studies have used ICU length of stay (LOS) as a composite measure of costs and resource use [6]. However, the relationship between the cost of care and the ICU LOS is imperfect [7–10]. Another complicating factor is that the total ICU costs are composed of both fixed and variable costs, of which fixed costs are estimated to constitute nearly 80% of ICU costs [11, 12].

The COVID-19 pandemic has changed the dynamics of ICU care significantly. Unfortunately, at the beginning of the pandemic, ICUs were unprepared for the surge of sick patients [13]. Global supply-chain disruptions have prompted healthcare organizations to change their focus from ‘just in time’ to ‘just in case’, i.e. ensuring effective critical care surge response, including consideration of ICU staffing models, having national or regional strategic reserves of personal protective equipment, devices, consumables, and pharmaceuticals [14, 15]. There is a need to implement cost-effective measures without compromising the safe delivery of value-based healthcare while maintaining a buffer for ‘just-in-case’ scenarios. Unfortunately, there is limited literature available to guide this, hence this multidisciplinary and multinational Delphi study that aimed to gather expert opinions and develop consensus to identify cost-effective interventions, the inTerventions tO Promote coSt-effectiveness In aDult intEnsive care units (TOPSIDE study).

## Methods

We followed ‘Guidance on Conducting and REporting DElphi Studies’ (CREDES) guidelines to plan and present the Delphi study results [16]. The National Healthcare Group (NHG) Domain-Specific Review Board (DSRB), Singapore approved the study with a waiver of informed consent due to the non-interventional, Delphi survey design (NHG DSRB reference number—2023/00414). Consent was implied when experts participated in the Delphi process by completing the online surveys.

### Purpose and rationale

Delphi techniques are widely used in healthcare to answer relevant questions where the research is limited, ethically/logistically difficult or the evidence is equivocal [17]. Previously, Delphi methods have been used in the ICU settings to reach a consensus on a standard set of ICU discharge criteria [18], to optimize clinically relevant drug-drug interactions [19], to develop a set of ‘top tips’ for good healthcare communication [20], to define competencies [21] or research priorities [22, 23]. Therefore, we sought to identify the interventions to promote cost-effectiveness using a modified eDelphi methodology.

### Expert panel and steering committee

Experts were identified from a convenience sample of multinational subject matter experts through peer recommendation. We used World Health Organization (WHO) endorsed categorization of countries according to Gross National Income (GNI) per capita [24]. Our definition of being an expert was defined as being a clinician (doctors, nurses and allied health professionals) practising in an ICU and/or an administrator with extensive ICU clinical and healthcare service management experience, all experts had at least 5-year post-graduate experience.

As with previously published guidelines, the decision regarding the number of experts was a pragmatic choice; a key consideration was to ensure good representation with qualified intensive care providers [25]. Patients and the public were not involved in the study. We formed a steering committee of 13 experts to guide the study and associated discussions (AK, JML, KCS, SR, MC, CB, ACM, RDS, SR, JPF, VMN, BKR, RCM). The steering committee comprised colleagues holding current or recent leadership positions in various national and international Intensive Care societies or were senior ICU clinicians, and/or senior researchers with at least 10-year experience. Due to the size of the steering committee, and the ability to blind the committee to the results prior to completion of each round it was decided to include steering committee members in the expert group given their international leadership roles and expertise in the subject matter. One member of the steering committee, AK, administered and coordinated the Delphi survey and therefore was the only member of the steering committee not to participate in the expert group.

### Description of the methods

The Delphi study was conducted from November 2022 to February 2023, followed by steering committee discussions in March-June 2023. Experts were given 3 weeks to respond to each round, with 2 reminders a week apart.

This was a modified Delphi survey with a succinct mixed qualitative and quantitative approach. The Delphi surveys were administered through an online Delphi survey platform (Welphi™, Decision Eyes, Lisbon Portugal), facilitating global expert involvement and streamlined collation of responses.

Round 1 consisted of an open-ended questionnaire. Experts were requested to propose interventions in four domains, following the structure set out by the US National Academy of Medicine in their approach to high-value healthcare [26]. The four domains are:

1. Foundational elements: Developing a culture of continuous improvement, through quality improvement methodology.
2. Infrastructure fundamentals: Implementing evidence-based care, information technology best practices, efficient care delivery, and the sustainable use of resources.
3. Care delivery priorities: Integrated and coordinated care delivery, through team-based approaches and shared decision-making.
4. Reliability and feedback: Embedding safeguards, anti-microbial stewardship and being transparent within the health service.

In the first Delphi round, experts were invited to list and provide comments regarding interventions that promote cost-effectiveness considering resource limitations, especially in the peri-pandemic era. Individual responses were collated and converted into a list of 60 interventions for the second round. Round two of the Delphi process was composed of quantitative as well as qualitative components. All the experts from round 1 were included in round 2, irrespective of whether they had responded earlier, as the first round was deemed exploratory in nature. The experts were able to see the questionnaire, along with anonymized responses from round 1 before scoring in round 2 (Additional file 1: Fig. S1). This provided an opportunity for them to revise their judgments based on comments from their peers. In round 2 the experts were requested to rate the proposed interventions on a 5-point Likert scale (ranging from strongly agree, agree, neutral, disagree to strongly disagree) along with an opportunity to offer optional narrative comments on each statement.

At the end of round 2, qualitative and quantitative data were collated. The items from round 2 were retained for round 3, which was quantitative in nature. The third round only included experts who had responded in the second round. As before, the experts were able to see the same questionnaire from the previous round, along with anonymized responses before re-scoring (Additional file 1: Fig. S2).

We adopted a dynamic approach regarding the number of rounds, with subsequent rounds incorporated if the steering committee deemed it necessary for further clarification [27]. As in previous studies, we observed that there were no changes in experts’ ratings between rounds two and three [28], and the steering committee decided that another round was not warranted. At the end of round 3 a list of 50 items was produced, in order to make this appraisable the steering committee drew this into a smaller list through combining topics into common themes and identifying high-impact items. This process was undertaken through iterative rounds of discussion and voting within the steering committee.

### Definition and attainment of consensus

We applied previously published consensus criteria for Delphi studies to the quantitative results of 3 rounds [25]. Interventions with over 70% consensus (scored as ‘strongly agree/agree’), which < 15% of respondents scored as ‘strongly disagree and disagree’, were recommended as cost-effective measures. Conversely, if over 70% of experts disagreed on an intervention (by scoring strongly disagree or disagree), with fewer than 15% agreement (strongly agree and agree), this intervention was considered as ‘undesirable’. An intervention was classified as achieving ‘no consensus’ if neither criterion was met. We also compared the list of interventions between rounds 2 and 3 to assess for impact of peer opinion among the experts.

These consensus items endorsed by the Delphi experts were subjected to iterative discussions by the steering committee through face-to-face interactions, online video meetings and electronic written discussions. Each steering committee member voted to select ten interventions that they deemed important. One steering committee member (AK) was excluded from final voting due to risk of bias in view of access to identifiable comments as the lead administrator of Delphi process. Items to be identified as ‘critical’ required consensus by more than 50% of steering committee members. This list of ‘critical’ interventions formed the basis of consensus statement and best practice considerations.

## Results

**Fig. 1:**
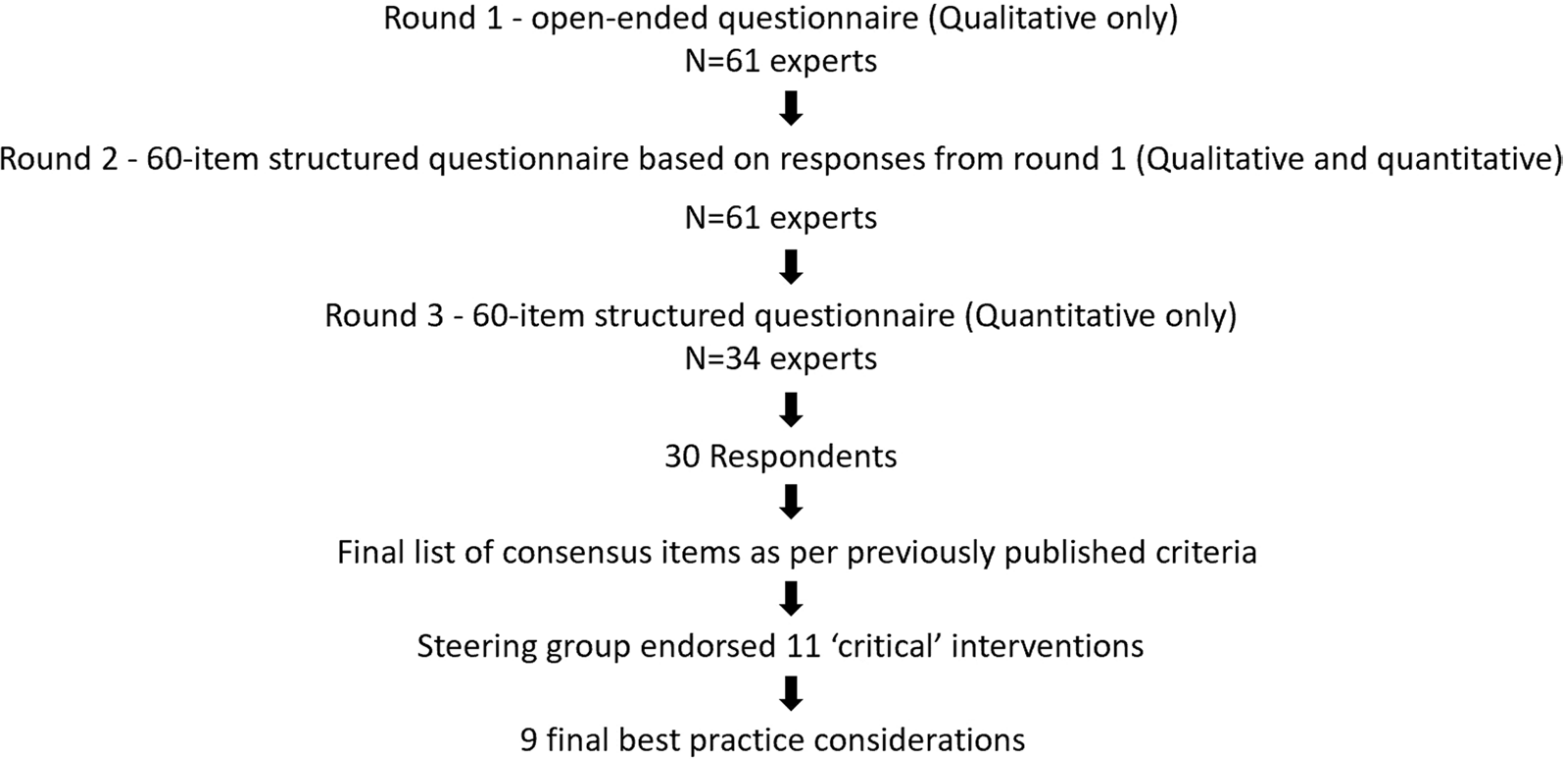
Flowchart to illustrate the stages of Delphi technique

A total of 61 experts participated in the Delphi study (Table 1). The response rates were 52% for round 1 (32/61), 56% for round 2 (34/61) and 88% for round 3 (30/34, the denominator being the number of experts who responded to round 2 of the Delphi survey), respectively (Fig. 1). Thirty experts participated in round 3 (Table 1, Additional file 2: Table S1).

**Table 1:** Baseline characteristics of experts

Panel composition (*n* = 61)	Physicians (*n* = 50, *82%*), Nurses (*n* = 5, *8%*), Medical Administrators (*n* = 3, *5%*), and Allied health—respiratory therapists and pharmacists (*n* = 3, *5%*)		
Country category, according to GNI per capita	Low- and middle-income countries (*n* = 26, *43%*)		High-income countries (*n* = 35, *57%*)
	Low and Low-middle-income countries *n* = 17—India *n* = 1—Bangladesh *n* = 1—Egypt *n* = 1—Nepal *n* = 1—Philippines *n* = 1—Sri Lanka	Upper middle-income countries *n* = 2—Malaysia *n* = 1—Thailand *n* = 1—Turkiye	*n* = 8—Singapore *n* = 8—USA *n* = 5—UK *n* = 3—Australia *n* = 2—Dubai *n* = 2—Italy *n* = 2—Portugal *n* = 1—France *n* = 1—Greece *n* = 1—Japan *n* = 1—New Zealand *n* = 1—South Korea
Experts who responded in round 3 (*n* = 30)	Physicians (*n* = 28, *93.3%*), Nurses (*n* = 2, *6.7%*)		
	Low- and middle-income countries (*n* = 12, *40%*)		High-income countries (*n* = 18, *60%*)
	*n* = 9—India *n* = 1—Nepal *n* = 1—Sri Lanka	*n* = 1—Turkiye	*n* = 4—UK *n* = 5—USA *n* = 2—Singapore *n* = 2—Australia *n* = 2—Italy *n* = 1—Dubai *n* = 1—New Zealand *n* = 1—South Korea
Steering committee (*n* = 13)	*n* = 4—India	NA	*n* = 3—USA *n* = 2—Singapore *n* = 2—UK *n* = 1—Australia *n* = 1—Italy

A total of 60 interventions were identified at the end of round 1 and the same 60 formed the basis for rounds 2 and 3. Of the 60 interventions, 50 interventions reached consensus criteria after round 2 and the same 50 items achieved consensus in round 3 as well (Table 2). Of note, 31 (51%) of these interventions achieved a consensus of 90% and above after round three.

**Table 2:** Interventions endorsed by Delphi study, categorized into nine sub-groups

	Infrastructure fundamentals	Care delivery priorities	Foundational elements	Reliability and feedback
Pandemic preparedness	(1) Optimize resource usage for pandemics	(2) Establish a multidisciplinary disaster/pandemic response team	(3) Audit compliance to best practices in a pandemic situation	
ICU Organization	(4) Standardize and establish governance of ICU setups (integrated or otherwise) at regional/ national levels (5) Availability of all the relevant experts including all laboratory services (6) Develop an international consensus report on the Green ICU concept (7) Counselling rooms with Hospital Information System accessibility within that room (8) Point of care diagnostics (9) Structural elements (Hospital level)^#^ (10) Structural elements (ICU design)^#^	(11) Champion-led team approach^#^ (12) Multidisciplinary patient care model (Multidisciplinary team consisting of a trained intensivist, a trained ICU nurse, physiotherapist, pharmacist, speech pathologist, etc.) (13) Early mobilization (14) Team building and support (15) Multidisciplinary approach to implementing end-of-life interventions focused on patient- and family-centric care	(16) Ensure institutional and ICU leadership support	(17) Provide access to cost (and charge) information for treating teams (18) Benchmark units at the National (or regional) level
Establishing and pursuing standards of care			(19) Quality audits to identify cost-reduction opportunities (20) Multidisciplinary Practice Evaluation Programs, involving a wide range of ICU professionals. Engagement with hospital administrators in setting quality indicators (21) Standardize practice through protocols/ care pathways and ongoing audits (22) Setup evidence-based standards of care, including interventions for which there is limited or no evidence (23) Establish standard ICU audit guidelines, adapted to local circumstances	(24) Critical incident report system (25) Regular surveillance and monitoring of the clinical practice with feedback to the ICU team
Resource optimization	(26) Dynamic staff roster to accommodate even distribution according to workload (27) Cost-effective sterilization practices (28) ICU equipment and devices related		(29) Audit of consumable usage (30) Reduce frequency of laboratory tests and radiological tests through Quality Initiatives (31) Rationalize transfusion practices	(32) Rationalize the use and having a stewardship approach towards critical resources
ICU/ HDU admission and discharge optimization		(33) Careful discharge planning (34) Appropriate use of ICU/ HDU resources		(35) Review of failed discharges/ ICU re-admission to identify opportunities for improvement (36) Audit of time taken for ICU admission from Emergency Department/ Wards
Expanding the scope of the ICU beyond the four walls of the ICU	(37) Develop step-down units and long-term care units (38) Appropriate remote monitoring in step-down units (39) Utilize tele-ICU to bring down ICU costs as well as support under-served areas	(40) ICU-team-led Outreach services on wards (41) Establish a multidisciplinary rapid response team led by ICU		
Competencies and training of staff	(42) Develop and Maintain Competency (43) Continuous training & education		(44) Audits of staff competency, training of staff	
Infection control measures				(45) Hand hygiene monitoring (46) Setup governance of antibiotic stewardship institutionally (47) Setup anti-microbial stewardship including Infectious diseases-ICU rounds (48) Healthcare acquired infections prevention
Electronic health records	(49) Electronic Health Records in ICUs			(50) Electronic health records to increase accountability

*HDU* high dependency unit, *ICU* intensive care unit

^#^Details in supplementary table 2

Ten interventions did not achieve Delphi consensus for prioritization (Table 3).

**Table 3:** Cost-effective interventions which did not achieve Delphi consensus for prioritization at this time

Cost-effective interventions which did not achieve Delphi consensus for prioritization at this time	Degree of endorsement (Strongly agree and agree) in round 3
*Infrastructure fundamentals*	
(1) Integrated ICU model preferable to Emergency Department-based ICU/Specialty-based ICUs	74% (However, ≥15% Strongly disagree and disagree)*
(2) Creating Critical Care Nurse Consultants, Physician Assistants as part of the critical care team	66%
(3) A combined ICU & HDU model	62%
(4) Low-cost wearable devices to replace the expensive commercial equipment for physiological monitoring	56%
(5) Opportunities to use artificial intelligence	55%
(6) Hand-held imaging devices such as ultrasound probes attached to smartphones	55%
(7) Surgical intermediate care unit as cost-saving alternative to ICU care	41%
(8) Use of disposable items over reusable	29%
*Care delivery priorities*	
(9) Post-intensive care outpatient clinics under the supervision of intensivists	63%
*Reliability and feedback*	
(10) Linking KPIs to physician/unit remuneration	41%

*HDU* high dependency unit, *ICU* intensive care unit

*One intervention, namely, “Integrated ICU model preferable to ED-based ICU/ Specialty-based ICUs” scored more than 70% agreement in both rounds; however, the disagreement was 15% and we counted this intervention as a “Cost-effective intervention which did not achieve Delphi consensus for prioritization”

The structured details of qualitative responses from rounds 1 and 2 are presented separately (Additional file 3: Table S2).

Thereafter, the steering committee endorsed 11 interventions that were identified as ‘critical’ by more than 50% of steering committee members. These interventions and experts’ comments were summarized as 9 final considerations for best practice (Box 1):

1. Adoption of multidisciplinary patient care model:
  o Multidisciplinary team should consist of skilled professionals with expertise in critical care (intensivist, ICU nurse, and allied health professional) and conduct daily rounds. Multidisciplinary care could promote early extubation, and early mobilization and expedite timely ICU discharge.
2. Development and maintenance of staff competency, and audit:
  o ICU staff should be supported in developing and maintaining critical care competency and skills to develop a continuous learning process. Development of core curriculum and structured training programmes for staff across all relevant professional groups, and mapping training onto that curriculum would further help with credentialling and ensuring standardization of ICU knowledge.
  o Additionally, staff competency should be regularly audited and reviewed with participation of all stakeholders. Consideration should be given for ongoing personal performance evaluation plan for each physician and end of year evaluation.
3. Development of step-down units and long-term care units:
  o Definition of ICU versus 'high dependency/ step-down/ long-term care units' should be established as agreed upon at regional/ national level. Consideration should be given to standard policies, good governance, and appropriately trained and skilled workforce.
4. Organization of tele-ICU services to bring down ICU costs as well as support under-served areas:
  o Telemedicine and remote review should be considered where access to physical presence of a trained ICU team is not possible. Any such advice should be based on a thorough assessment based on clinical, laboratory, and radiological data. A professional relationship between the remote expert and the on-site team with the ability to provide on-site visits or patient transport if required, should be considered. There should be governance and feedback process to oversee the service.
5. Adoption of dynamic staff roster to accommodate even distribution according to workload:
  o ICUs should adopt a rational staffing approach introduced with flexible and even distribution of staff according to the workload, to avoid burn out and exhaustion. However, this approach should also consider the challenge of work-life balance and staff retention.
6. Implementation of end-of-life (EOL) interventions:
  o Consideration should be given to multidisciplinary approach focused on patient- and family-centric EOL care, which could avoid unnecessary ICU admissions, avoid prolonged ICU stays, and challenges in withdrawing the already instituted care, thereby reducing costs through reduction in futile treatment. The specific skills and expertise required for this aspect of practice should be part of the core curriculum for intensivists and other critical care professionals.
7. Adoption of early mobilization:
  o Early mobilization provided by the designated ICU physiotherapist and nursing team would help in enhancing ICU recovery in patients at the earliest opportunity and can facilitate early discharge.
8. An international consensus effort on the ‘Green ICU’ concept:
  o establish consensus on minimising the environmental impact of ICU, through factors such as the utilization of energy efficient lighting, and recycling of non-contaminated plastic waste.
9. Implementation of audits to promote a culture of continuous quality improvement:
10. Multidisciplinary Practice Evaluation Programs should involve as many ICU professionals as possible, collaboration with nursing staff, and engage with administration in setting quality indicators.
11. Standard ICU audit guidelines, adapted to local circumstances should be implemented (e.g. Guidelines for Provision of Intensive Care Services by Intensive Care Society [29]).
12. Frequency of laboratory and radiological tests should be optimized through quality improvement methodology.

**Box 1:** Consensus statement and considerations for best practice interventions to promote cost-effectiveness in adult intensive care units

(1) Adoption of multidisciplinary patient care model
(2) Development and maintenance of staff competency, and audit
(3) Development of step-down units and long-term care units
(4) Organization of tele-ICU services to bring down ICU costs as well as support under-served areas
(5) Adoption of dynamic staff roster to accommodate even distribution according to workload
(6) Implementation of end-of-life (EOL) interventions
(7) Adoption of early mobilization
(8) An international consensus effort on the ‘Green ICU’ concept
(9) Implementation of audits to promote a culture of continuous quality improvement

## Discussion

We conducted a multidisciplinary and multinational eDelphi survey followed up with iterative discussions among the steering committee, to gather expert consensus to identify interventions that promote cost-effectiveness in adult ICUs. Final best practice considerations included adoption of a multidisciplinary ICU model, strong focus on staff training and competency assessment, and audits to objectively promote a culture of continuous improvement. These aim to ensure high quality critical care services both inside and outside ICUs. Additionally, implementation of a dynamic staff roster, multidisciplinary approach to implementing EOL care, early mobilization and promoting international consensus efforts on the ‘Green ICU’ concept were endorsed. These findings represent, to the best of our knowledge, the first time a Delphi study has been used to address the complex issue of cost-effectiveness with global appeal involving experts from across varied healthcare systems, extending beyond regional and national boundaries. These best practice considerations are designed to be equally applicable to low-income as well as middle-high-income countries, albeit requiring tailoring according to local resources.

Many of the proposed best practice considerations are supported by previous studies suggesting outcome benefits including actual or hypothetical cost benefits, though cost-effectiveness was not studied, e.g. case of multidisciplinary team model [30–32]. One systematic review of cost-related impacts from utilizing respiratory therapists to deliver care showed both direct and indirect cost reductions, which were achieved through protocol utilization, specialized expertise, and autonomous decision-making [32].

Best practice consideration to develop step-down units, long-term care units, and tele-ICUs comes with an emphasis on ensuring appropriate staffing, adequate resources, and robust governance process. On the other hand, previously published literature is equivocal with regards to endorsing ICU-level interventions provided by other specialties (e.g. surgical intermediate care units, emergency department-based ICU) and manned by non-ICU teams, despite the availability of positive studies [5, 33–36]. This hesitancy may be due to concerns regarding quality of care being provided by non-ICU teams and highlights the need to ensure standardization irrespective of clinical team involved.

Similarly, audits to promote a culture of continuous improvement [37–40], multidisciplinary team approach to implementing EOL care [41, 42], and early mobilization [43] have shown cost benefits. Multidisciplinary team approach comprising physicians, clinical nurse specialists and/ or palliative team focused on improving communication with patients and patients' families at the end-of-life reduced LOS and lowered ICU costs and significantly improved nurse-assessed quality of dying [41, 42]. A systematic review comprising twenty-three randomized control trials involving 2308 critically ill patients showed that early mobilization decreased the incidence of ICU acquired weakness at hospital discharge, increased the number of ventilator-free days during hospitalization, and increased the discharged-to-home rate; as well as non-significant improvement in mortality (28-day, ICU, and hospital). However, substantial heterogeneity among the included studies, and the low quality of the evidence, warrants caution. Our Delphi survey adds weight of expert consensus to the existing evidence.

These consensus statements hold even more importance in the post-pandemic era of ‘just-in-case’, with competing priorities for the finite resources available. The WHO has recently declared an end to COVID-19 as a global health emergency in May 2023. Few of the interventions proposed by Delphi experts were categorized under pandemic preparedness (optimization of resource usage during the pandemic setting, establishment of a multidisciplinary disaster/pandemic response team and emphasis on audit to ensure compliance to best practices in a pandemic situation). As this Delphi survey was conceived and conducted during the pandemic period, experts’ emphasis on pandemic preparedness is not surprising. However, these considerations are equally applicable to the non-pandemic times.

Interestingly, several of the proposed interventions did not reach consensus despite some proposed benefits in previous studies, e.g. low-cost wearables combined with artificial intelligence for physiological monitoring, point-of-care diagnostic and imaging devices, and use of disposable items over reusable [44]. Possible reasons could be that such interventions need upfront expenses with unclear cost recovery downstream, and lack of confirmatory evidence of their efficacy in relevant clinical settings.

Our Delphi study has several limitations. We sought to balance the views of established experts with publication and other markers of expertise in sustainable and cost-effective care with broader, lived expert experience of ICU and thus seek to ensure the views were more generalizable. While these individuals had many years of ICU experience, as a necessary pragmatic need to maintain an effective number of Delphi participants we cannot guarantee that they fully considered every issue of this admittedly broad topic, e.g. regional variations in practice due to regulatory issues surrounding ICU setups, availability of drugs and devices. Although we had a reasonable geographic (we note a lack of experts from African countries) and national income-level (excluding low-income countries) spread of experts, the opinions may not represent those from areas which are not covered. However, the experts did represent a good mix of intensive care professionals from low-middle-income, and high-income countries.

Secondly, we present percentages for agreement. However, our Delphi survey was not designed to understand the differences in case of non-agreement, any further analysis in form of a mediation analysis will not be possible given the dimensionality of our dataset. The regression models for a mediation analysis would not pass the goodness-of-fit test.

Thirdly, there was no patient involvement as well as no distinct stakeholder groups. This may have limited the ability of our findings to fully represent a holistic perspective and failure to highlight any disparate views. Our relatively small number of stakeholders and higher attrition in non-physician categories precluded such groupings (Table 1). However, even in a multiple-panel study consisting of different stakeholder groups, it is difficult to ascertain what weightings should be given to each group and there is no current guidance on this.

Additionally, our Delphi study asked the experts to categorize proposed interventions into four sub-categories, following principles of high-value health care which have previously shown promise in lowering costs [26]. These principles however were not critical care specific, were derived from one high-income nation and were opinions only from senior hospital administrators.

Lastly, we had a response rate of just over 50% between round 1 and round 2 as well as higher attrition in non-physician categories. In view of the intention to achieve a global distribution of the experts, we were dependent on the peer recommendation to identify these experts. Response rates are often a challenge in studies such as this, and responders are more likely to have an interest and self-assessed expertise in the topics covered. Non-responders might have had limited interest in participating in the Delphi survey, or were unable to due to time pressure and competing priorities. However, we were able to achieve a response rate of 88% in the third round, further strengthening our view that we selected those with greatest interest and self-assessed expertise for the final recommendations. Attrition bias can occur when the participants that do not respond in subsequent rounds have different views from their peers who continue to participate. However, when we compared the round 3 and round 2 results, we observed no variation in opinions and a retention of > 80% which is considered satisfactory as per published literature [22].

While we have produced a number of best practice considerations, how they should be implemented and what metrics be used to assess their effective implementation require further work. We believe that these best practice considerations should be equally applicable to low-income as well as middle-high-income countries; albeit need tailoring according to local resources. Validation of these proposals by additional experts, involvement of more international scientific societies and involvement of patients and families are important next steps. Future research should focus on the selection of appropriately bundled interventions, tailored to regional needs and implemented under an appropriate framework of metrics.

## Conclusion

We present consensus statement and best practice considerations of interventions to promote cost-effectiveness in adult ICUs. The best practice considerations include adoption of a multidisciplinary ICU model of care, focus on staff training and competency, ongoing quality audits, implementation of dynamic staff rostering, multidisciplinary approach to implementing EOL care, early mobilization and promoting international consensus efforts on Green ICU concept. Stakeholders such as government bodies and professional societies must lead the efforts to identify locally applicable specifics with the available resources, while working within these best practice considerations.

### Supplementary Information

**Additional file 1: Fig.S1** The experts were able to see all the anonymized comments from the previous round for each of the interventions, by clicking on the ‘details’ section. **Fig. S2.** The experts were able to see all the anonymized comments from the previous round for each of the interventions, by clicking on the ‘details’ section.

**Additional file 2: Table S1.** Details of experts who participated in Round 3 (*n* = 30).

**Additional file 3: Table S2.** Round 1 and 2, qualitative results.

## Data Availability

The datasets used and/or analysed during the current study are available from the corresponding author on reasonable request.

## Abbreviations

CREDES: Conducting and REporting DElphi studies
DSRB: Domain-Specific Review Board
EOL: End of life
GNI: Gross national income
ICU: Intensive care units
LOS: Length of stay
WHO: World Health Organization
