# Correction to: Interventions to promote cost-effectiveness in adult intensive care units: consensus statement and considerations for best practice from a multidisciplinary and multinational eDelphi study

**DOI:** 10.1186/s13054-024-04888-1

**Published:** 2024-04-12

**Authors:** Amit Kansal, Jos M. Latour, Kay Choong See, Sumeet Rai, Maurizio Cecconi, Carl Britto, Andrew Conway Morris, Raymond Dominic Savio, Vinay M. Nadkarni, B. K. Rao, Rajesh Mishra

**Affiliations:** 1 Department of Intensive Care Medicine, Ng Teng Fong General Hospital, Jurong Health Campus, National University Health System, Singapore, Singapore; 2 School of Nursing and Midwifery, Faculty of Health, University of Plymouth, Plymouth, UK; 3 Division of Respiratory and Critical Care Medicine, Department of Medicine, National University Hospital, Singapore, Singapore; 4 Intensive Care Unit, Canberra Hospital, Canberra, Australia; 5 Department of Biomedical Sciences, Humanitas University, Via Rita Levi Montalcini 4, 20072 Pieve Emanuele, Milan, Italy; 6 Division of Critical Care, Department of Anesthesia, Critical Care and Pain Medicine, Boston Children’s Hospital, Boston, USA; 7 Division of Anaesthesia, Department of Medicine, University of Cambridge, Cambridge, UK; 8 Critical Care Services, Apollo Proton Cancer Center, Chennai, India; 9 Department of Anesthesiology, Critical Care, and Pediatrics at the Children’s Hospital of Philadelphia (CHOP), University of Pennsylvania Perelman School of Medicine, Philadelphia, USA; 10 Department of Critical Care Medicine, Sir Ganga Ram Hospital, New Delhi, India; 11 Shaibya Comprehensive Care Clinic, Ahmedabad, India; 12 IRCCS Humanitas Research Hospital, Via Manzoni 56, 20089 Rozzano, Milan, Italy; 13 Department of Nursing, Zhongshan Hospital, Fudan University, Shanghai, China; 14 John V Farman Intensive Care Unit, Addenbrooke’s Hospital, Cambridge University Hospitals NHS Foundation Trust, Cambridge, UK

**Correction to: Crit Care (2023) 27:487** 10.1186/s13054-023-04766-2

Following publication of the original article [1], the authors identified an error in the author name of Maurizio Cecconi.

The incorrect author name is: Maurizo Cecconi

The correct author name is: Maurizio Cecconi

The Funding section currently reads:

**Funding**

The study was not funded.

The Funding section should read:

**Funding**

ACM is funded by the UK Medical Research Council via a Clinician Scientist Fellowship (MR/V006118/1).

The author group and Funding section has been updated above and the original article [1] has been corrected.
