# Supplementary material for: Interventions to promote cost-effectiveness in adult intensive care units: consensus statement and considerations for best practice from a multidisciplinary and multinational eDelphi study
Source: Crit Care. 2023 Dec 11;27:487. doi: 10.1186/s13054-023-04766-2 (PMC10712165; doi:10.1186/s13054-023-04766-2)
Supplement: Supplementary file 1 — Additional file 1: Fig.S1 The experts were able to see all the anonymized comments from the previous round for each of the interventions, by clicking on the ‘details’ section. Fig. S2. The experts were able to see all the anonymized comments from the previous round for each of the interventions, by clicking on the ‘details’ section. [file 13054_2023_4766_MOESM1_ESM.docx]

**Interventions to promote cost-effectiveness in adult Intensive care units: consensus statement and considerations for best practice from a multidisciplinary and multinational eDelphi study**

**Supplementary Figures 1 and 2 (B & W)**

| **Supplementary Figure 1** |
| --- |
| 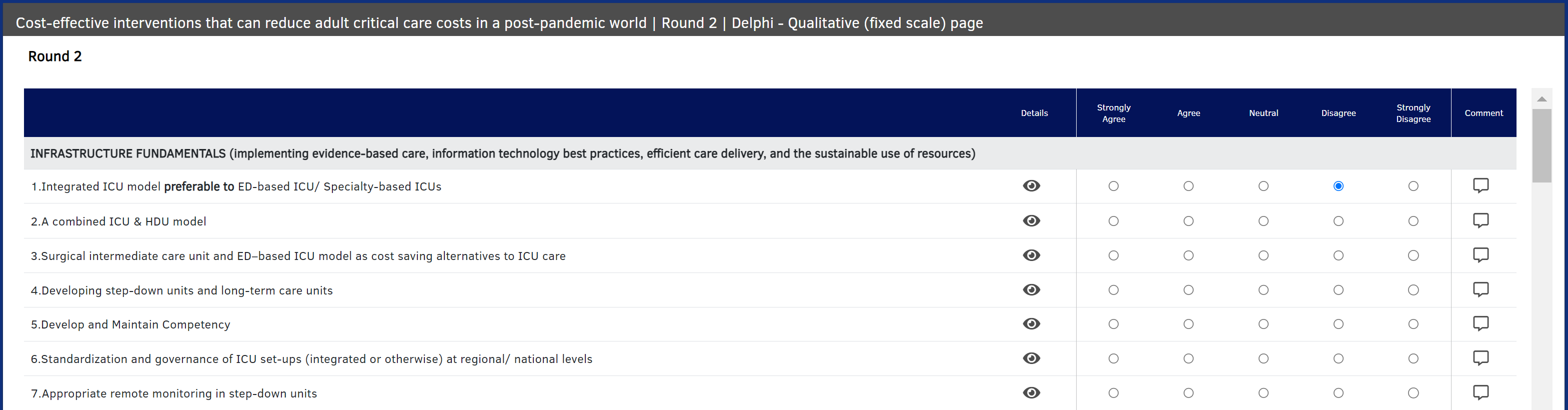 |
| 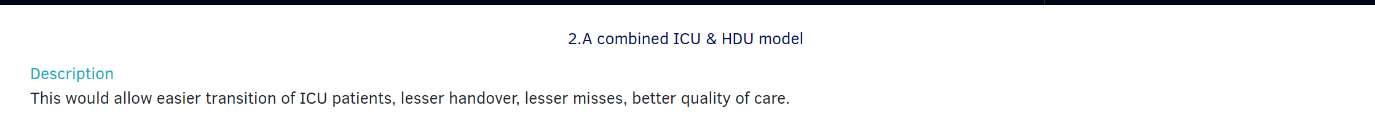 |
| Figure 1 - The experts were able to see all the anonymized comments from the previous round for each of the interventions, by clicking on the ‘details’ section |

| **Supplementary Figure 2** |
| --- |
| 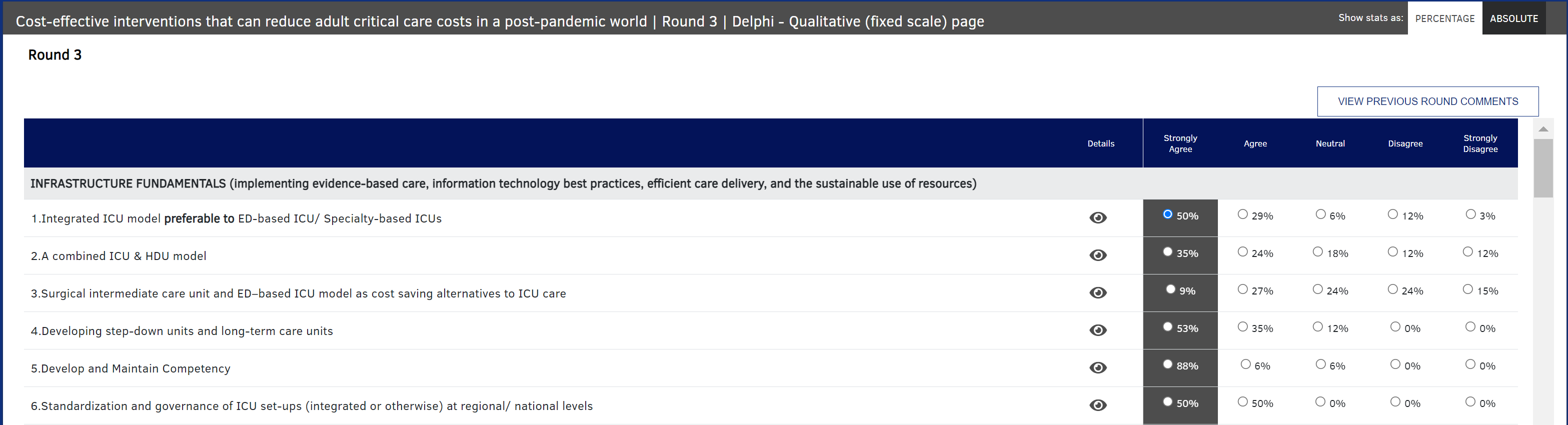 |
| 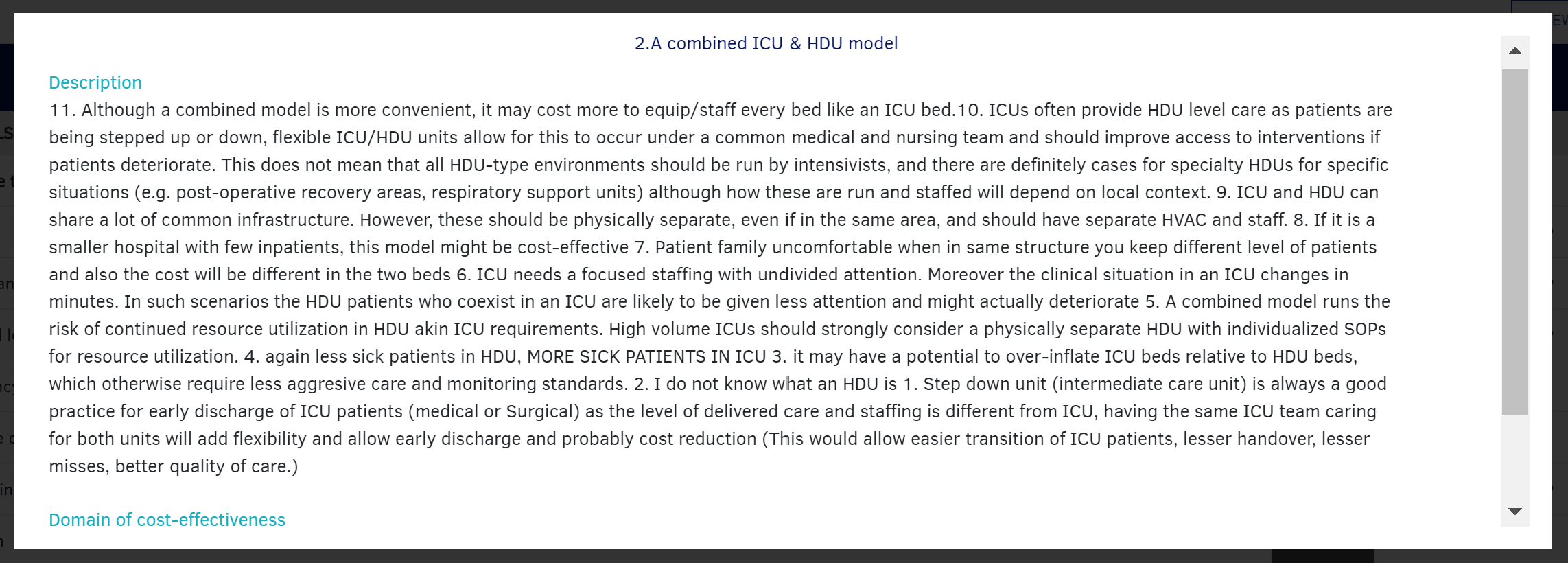 |
| Figure 2 - The experts were able to see all the anonymized comments from the previous round for each of the interventions, by clicking on the ‘details’ section |
