# Supplementary material for: Interventions to promote cost-effectiveness in adult intensive care units: consensus statement and considerations for best practice from a multidisciplinary and multinational eDelphi study
Source: Crit Care. 2023 Dec 11;27:487. doi: 10.1186/s13054-023-04766-2 (PMC10712165; doi:10.1186/s13054-023-04766-2)
Supplement: Supplementary file 2 — Additional file 2: Table S1. Details of experts who participated in Round 3 (n = 30). [file 13054_2023_4766_MOESM2_ESM.docx]

**Interventions to promote cost-effectiveness in adult Intensive care units: consensus statement and considerations for best practice from a multidisciplinary and multinational eDelphi study**

**Supplementary Table 1**: Details of experts who participated in Round 3 (n=30)

| **Country** | **Name** | **Affiliation** |
| --- | --- | --- |
| Australia | Prof Bala Venkatesh | MBBS FCICM |
|  | Dr Sumeet Rai | MBBS EDIC FCICM |
| Dubai | Dr Ashraf Elhoufi | MS MRCP FRCP EDIC |
| India | Dr Ashish Bhalla  Dr BK Rao  Dr Manish Bharti  Dr Pradeep Rangappa  Dr Rajesh Chandra Mishra  Dr Raymond Dominic Savio  Dr Sharmili Sinha  Dr Sivakumar M N  Dr Sumit Ray | MD  MD FICCM  MBBS DA MD FNB EDIC  MBBS DNB EDIC FCICM  MD FNB EDIC FCCM FICCM  MD DM EDIC FICCM  MBBS MD DNB  MBBS DA DNB IDCCM EDIC FICCM  MBBS MD |
| Italy | Adj Prof Alessandro Galazzi  Prof Maurizo Cecconi | PhD MSN CCRN  MBBS FRCA FFICM |
| Nepal | Prof Subhash Prasad Acharya | MBBS MD |
| New Zealand | Dr Ross Freebairn | FCICM |
| Singapore | Dr Amartya Mukhopadhyay  Dr Kay Choong See | MBBS FRCP MPH  MBBS MPH |
| South Korea | Prof Gee-Young Suh | MD PhD |
| Sri Lanka | Dr Anushka Mudalige | MD |
| Turkiye | Prof Arzu Topeli | MD |
| UK | Dr Ajay Gupta  Dr Andrew Conway Morris  Prof Jos M Latour  Prof Louise Thwaites | MBBS MD PhD  MBBS FFICM PhD  RN PhD  MBBS MRCP MD DMSMed MLCOM |
| USA | Dr Carl Britto  Dr Craig Coopersmith  Dr Javier Perez Fernandez  Prof Mitch Levy  Prof Vinay M. Nadkarni | MBBS DPhil  MD  MD, FCCM, FCCP  MD  MD MS FCCM |
