# Supplementary material for: Interventions to promote cost-effectiveness in adult intensive care units: consensus statement and considerations for best practice from a multidisciplinary and multinational eDelphi study
Source: Crit Care. 2023 Dec 11;27:487. doi: 10.1186/s13054-023-04766-2 (PMC10712165; doi:10.1186/s13054-023-04766-2)
Supplement: Supplementary file 3 — Additional file 3: Table S2. Round 1 and 2, qualitative results. [file 13054_2023_4766_MOESM3_ESM.docx]

**Interventions to promote cost-effectiveness in adult Intensive care units: consensus statement and considerations for best practice from a multidisciplinary and multinational eDelphi study**

**Supplementary Table 2: Round 1 and 2, qualitative results**

| **Pandemic preparedness** | |
| --- | --- |
| Infrastructure fundamentals - implementing evidence-based care, information technology best practices, efficient care delivery, and the sustainable use of resources | |
|  | 1. Optimize resource usage during pandemic setting, e.g.,  **Round 1** - Implement evidence-based workflow / protocol. E.g., newer drug prescribing protocol; use of remote monitoring for ICU equipment (e.g. ventilators), use of walkie-talkie systems to communicate with patients nursed in Isolation rooms to avoid staff from frequently entering patient's room unnecessarily, avoiding wastage of precious PPEs  **Round 2** - No one can be fully prepared for disasters at this scale. However, the COVID pandemic has demonstrated the need of preparation for mass-casualty/mass-illness situations; clear plans for escalation and identified ways of securing staff, strategic stockpiles of resources (ventilators, basic drugs, fluids) as well as regional intelligence on stock levels, mutual aid for sharing of resources (and transfer of patients to load-level) This requires regional level ICU coordination and cooperation (which also needs to be funded). Although of course the goal would be to optimize resources at all times, not just during a pandemic. |
| Care delivery priorities – integrated and coordinated care delivery, through team-based approaches and shared decision-making | |
|  | 2. Establish a multidisciplinary disaster/pandemic-response team –  **Round 1** - The team will debrief in the aftermath of the pandemic, update the policies / evidence-based practices regularly and will also be responsible to disseminate the updated info.  **Round 2** - Utilization of existing teams to create a multi-disciplinary team may be more cost-effective. |
| Foundational elements – Developing a culture of continuous improvement, through quality improvement methodology | |
|  | 3. Audit compliance to best practices in pandemic situation –  **Round 1** - processes should be in place for audit of workflow / protocol, manpower/ resource distribution and monitoring drug utilization and optimization  **Round 2** - Compliance to best practices can be achieved by having a regional or national ICU outcomes audit system, and this can provide important data for both individual units and policy makers at regional and national level. |
| **ICU Organization** | |
| Infrastructure fundamentals - implementing evidence-based care, information technology best practices, efficient care delivery, and the sustainable use of resources | |
|  | 4.Standardization and governance of ICU set-ups (integrated or otherwise) at regional/ national levels:  **Round 1** - consensus should be established regarding what constitutes 'ICU' and how levels below that can be defined. Some of these may be speciality specific (e.g. Acute Respiratory Units for patients with exacerbated COPD/asthma not requiring intubation) or more generic 'high dependency units' with staffing and equipment lower than is required in ICU. For organizations some solutions may suit better and others will not, therefore a menu of choices is required. However, ensure UNIFORM treatment protocols across attached ICUs  **Round 2** - Establishing uniform treatment protocols across critical care units sound appealing, may result in a better standardization of care and avoid significant deviations based on personal opinions which may not be evidence-based. However, implementing this in the setting of incredibly disparate resources may be impractical, therefore, important to keep within the context of local logistics. |
|  | 5.Availability of all the concerned experts including all laboratory services  **Round 1** - Versus the need for send-away samples to national or regional laboratories -which will lead to slower decision-making  **Round 2** - ICU care involves multi-professional team and associated infrastructure and support services. If this is not done, inefficiencies might occur leading to more expenses or worse outcomes (or both). Laboratory specialities, most especially microbiology, have important inputs into ICU and should be readily available for consultation at all times of the clock - microbiology should ideally provide daily input into patients whilst haematology/biochemistry/immunology can usually be consulted on an 'as-required' basis.  Closed ICUs are proven to be the safest and most effective but need to work closely with parent specialties of patients especially when dealing with rarer conditions; effective partnership with intensivist leading the key decisions in consultation with parent specialties is the optimal organisation. Some resources may be "remote" but still available by telemedicine and tele-support. |
|  | 6.An international consensus report on Green ICU concept could be developed. |
|  | 7.Counselling rooms with Hospital Information System accessibility within that room: **Round 1** - To facilitate family meetings, enabling sharing patient information first hand with patient relatives, helps them understand the nature of illness and further care plans easily  **Round 2** - family consultation rooms are vital, however, we should move away from the concept of "room" and into concept of "space"; can be physical or virtual. Access to EHR is useful but not essential |
|  | 8.Point of care (POC) diagnostics:  **Round 1** - POC ultrasound and rapid diagnostic tests (e.g., point of care ABG machines, microbiological essays, coagulation testing) can help to optimize the management plans earlier and help more precision medicine. However, it may be expensive technology up-front with more cost-effective outcomes in long term  **Round 2** - Access to validated, quality assured POC diagnostics is vital in ICU when patients may change on a minute-by-minute basis and treatment delay can be fatal. The availability of bedside ABG & USG have clearly revolutionized care and will be expected to bring about cost effectiveness. However, individual pricing, ICU surcharges, indiscriminate usage are factors that need to be looked into as well as have to ensure quality assurance. |
|  | 9.Structural elements (Hospital level):  **Round 1** - Building ICUs in one floor so as to maximize utilisation of personnel strength; instead of building many ICUs and ICU beds, each hospital bed or room could be turned to a well-equipped and well-staffed ICU bed or room!  **Round 2** - ICUs need to be integrated into hospitals, with ready access to major sources of patients (ED, operating theatres) and diagnostics (radiology) - and indeed ready access to these areas should interventions be required. ICU patients should be cohorted in a single location. Where possible ICUs should be purpose built, with areas identified for overflow in case of surge in cases which should meet minimum ICU standards of space and facilities.  However, this will be a wasted expense and wasted space to make every single new bed compatible for ICU or floor) |
|  | 10.Structural elements (ICU design):  **Round 1** - Room setup should include variable set up for positive or negative pressure rooms, single rooms with less risk of cross contamination, be able to care for a varied range of HDU and ICU patients, designed to care for two patients with ease, but at the same time able to convert to a single room for an ICU patient. Room design should focus on natural light (for delirium prevention) and ability to control optimal temperature in the ICU rooms to reduce unnecessary heating and cooling cost. ICU design set up such that a reasonable number of beds can be divided into pods and can be looked after by a separate team.  **Round 2** - Architectural standards for ICUs are vital, should ensure adequate bed-space for all organ support machines, appropriate monitoring, access to power and medical gas supplies without impeding access to the patient, and processes for infection control (adequate ventilation, appropriate positive/negative pressure rooms to deal with infectious/vulnerable patients), suitable air cleaning to reduce risk of airborne infection spread (as demonstrated during COVID) which is better built into the building rather than deployed ad-hoc as portable devices. It is also important to ensure nursing/medical/allied health staff have adequate work space for near-patient admin (tables, computers, printers) as well as areas away from patients for administration and meetings/education. Family consultation rooms , of adequate size and number as well as waiting areas for family members are also an important consideration. Staff also need rest areas to allow time away from patients and for food/hydration, which need to be large enough to accommodate a significant proportion of the staff on shift at any given time. Changing areas and showering facilities are needed to allow changing into and out of work clothes 8.I am not sure what type of design elements would be helpful for cost saving - however most of these are helpful for quality of care.  However, the ICUs will look so remarkably different between countries (or states or hospitals or healthcare systems) that it might be difficult to standardize beyond a basic level. |
| Care delivery priorities – integrated and coordinated care delivery, through team-based approaches and shared decision-making | |
|  | 11.Champion-led team approach:  **Round 1** - Critical care should be provided by a team led by full-time champion, with openness to obtain appropriate referrals from other specialities which are likely to have an impact on early recovery. However, avoid unnecessary routine opinions that could result in excess cost. Overall, important to avoid misdiagnosis, or overdiagnosis. Having an accurate diagnosis early on would avoid delays in care, and unintended treatment/ cost.  **Round 2** - Intensivist can have any background basic specialty but once working in ICU it should be a dedicated 24/7. Intensive care is full time specialty, transition can take time. An intensivist has a deeper understanding of the existing physiological state of the patient having spent greater time at the bedside. |
|  | 12.Multi-disciplinary patient care model (Multidisciplinary team consisting of a trained intensivist, a trained ICU nurse, physiotherapist, pharmacist, speech pathologist, etc.):  **Round 1** - Daily multi-disciplinary ICU rounds and use of check-lists may facilitate robust planning for the patient and effective execution at the earliest opportunity, there-by early recovery can be expected with cost reduction e.g. reduced LOS.  **Round 2** - Important that patients are managed by skilled professionals with expertise in critical care -this is applicable across all professional groups and not just doctors. Multidisciplinary care could also speed up ICU discharge, early extubation, early mobilization. |
|  | 13.Early mobilization:-  **Round 1** - Early mobilization as a priority, provided by the designated ICU physiotherapist team helps in enhancing ICU recovery in patients at the earliest opportunity.  **Round 2** - Conceptually this makes sense, can facilitate early discharge and can definitely reduce ICU costs. Having said that, the data to support this are fairly weak. |
|  | 14.Team building and support:-  **Round 1** - Efforts should be taken to maintain the morale of the team by providing psychological support, offering debrief opportunities, valuing the contribution of all members is valued.  Continuous staff turnover increases costs apart from losing important resources.  **Round 2** - Simulation-based work and wider team building can be of benefit, similarly improved inter-personnel rapport in this highly chaotic and emotional environment can help.  Recruiting and retaining qualified and trained staff is essential to maintain a high quality services, But with the current Economic crisis and the high demand in certain countries/services (high income, government Vs private) it may be very difficult to retain qualified/ trained staff. |
|  | 15.Multidisciplinary approach to implementing end-of-life (EOL) interventions focused on patient- and family centric care:-  **Round 1** - Poor EOL care will result in un-necessary ICU admissions, un-necessary long stay with many problems in withdrawing the already instituted care, driving futile treatment costs. It would be useful to have EOL discussions done as early as in emergency departments.  **Round 2** - This is a key part of ICU care, and should not be seen as a failure of ICU but rather an appropriate outcome for patients who cannot be salvaged. This requires specific skills and expertise which should be part of the core curriculum for intensivists and critical care professionals, however access to palliative care services can also be useful in these circumstances.  EOL is sometimes difficult to implement because of many obstacles, part of it may be cultural, religion, and/ or the VIP culture. |
| Foundational elements – Developing a culture of continuous improvement, through quality improvement methodology | |
|  | 16.Institutional and ICU leadership support:-  **Round 1** - Support from hospital CEO/HOD, towards a culture of continuous improvement.  **Round 2** - ICU needs leadership as much as any other part of the hospital, and leaders need the time and resources to be able to lead effectively. Leadership is important for driving forward care quality and also ensuring accountability for outcomes and actions. |
| Reliability and feedback - embedding safeguards, anti-microbial stewardship and being transparent within the health service | |
|  | 17.Having access to cost (and charge) information:–  **Round 1** - Clinicians should know the costs of medical services (blood tests, diagnostics).  **Round 2** - This may be helpful in avoiding waste. Physician working in ICU particularly senior staff and HOD must have an access to cost of treatment and proper analysis of the details of why cost is increased and methods to decrease cost. Have to be careful regarding manipulation by hospital administrators and ensure that the information provided is not frivolous. |
|  | 18.Benchmark units at National (or regional) level:–  **Round 1** - Hospitals and ICUs should agree on the outcome measures, publish against national benchmark performance. Audits of critical care outcomes can help benchmark units and identify units which may need additional assistance (and indeed those who are performing above the norms and may be suitable to emulate). These will drive improved performance and quality improvement projects and improve care and reduce cost. Outcomes which can be looked at include mortality (risk adjusted), time to admission, severity of organ failure on admission, discharges outside office hours, hospital-level cardiac arrest/peri-arrest rates, delayed discharge (i.e. from when patient is fit for discharge to when they leave the unit) and health care associated infections - e.g. Rate of VAP/ CLABSI/ Surgical Site infections as well as Hospital falls/Pressure injuries. It is vital that such audits adjust for the unit organization, patient case mix to ensure like-for-like comparisons as otherwise findings may be misleading. |
|  | |
| **Establishing and pursuing standards of care** | |
| Foundational elements – Developing a culture of continuous improvement, through quality improvement methodology | |
|  | 19.Encourage audits to identify cost reduction opportunities  **Round 1** - Auditing compliance to medication related guidelines, care bundles to reduce complications [including Healthcare associated infections (HAI)] for ICU patients help drive cost down. Audits of sedation practices and weaning protocols, ensuring that patients are liberated from ventilatory supports at the earliest reducing the financial burden both from duration of stay and also implications from HAI  **Round 2** - It is vital that the quest for efficiency is not at the expense of quality - where products are substituted it should be with evidence of equivalent effectiveness, where treatments are removed it should be where there is evidence of harm or evidence of no effect (and with exceptions when carefully argued for- this would need some form of oversight). Where such evidence does not exist, it is the responsibility of the ICU community to undertake the research to establish or refute the utility of a given therapy. |
|  | 20.Multidisciplinary Practice Evaluation Programs, involve as many ICU professionals as possible, collaboration with nursing staff, Engage with administration in setting quality indicators  **Round 1** - Risk prevention costs less than problem solving. However, quality improvement interventions require resources (personnel, time) to be effectively applied.  **Round 2** - Multidisciplinary team acceptance and administrative will are essential for a successful quality program in ICU. |
|  | 21.Better standardization of practice though protocols/ care pathways and ongoing audits  22.Setting of evidence-based standards of care, including interventions for which there is limited or no evidence.  23.Using standard ICU audit guidelines, adapted to local circumstances.  **Round 1** - International/ national/ regional guidelines on minimal requirements in the organization and practice of intensive care medicine (e.g., General Standards for Provision of Intensive Care (GPICS) produced by the UK Intensive Care Society). Guidelines need to be streamlined / modified by individual institutions to adapt to their specific requirements, e.g., early tracheostomy in appropriate patients can reduce ICU stay if weaned earlier, making a huge saving for the appropriate patients - for instance in trauma, neurology patients.  Evaluation of unit practices against these national standards, looking specifically for areas where expensive non-evidence-based treatments are being used.  **Round 2** - Standard guidelines should ensure minimum expected level of care, but has to be tailored to local infrastructure and technology. These documents needs to be updated regularly and needs wider acceptance amongst the consultants. |
| Reliability and feedback - embedding safeguards, anti-microbial stewardship and being transparent within the health service | |
|  | 24.Critical incident report system  **Round 1** - Critical incident report system in operation can identify and allow review of critical events for future learning, and take necessary Corrective And Preventive Action (CAPA), to prevent further occurrence, there by reducing negative impacts and health costs that stem from them.  **Round 2** - This needs to occur in a culture of openness and without blame to encourage reporting and ensure lessons are learned by all involved. This requires administration support to avoid punitive actions and consider them as corrective actions; which will simply prevent staff from reporting and will encourage the cover up attitude. |
|  | 25.Regular surveillance and monitoring of the clinical practice with feedback to ICU team  **Round 1** – Morbidity and mortality meetings, regular clinical governance meetings to analyse the data on healthcare associated infections & quality indicator data for the unit etc, and continuous evaluation and feedback to multi-disciplinary personnel, can reduce cost, by revealing gaps in practices, breaches in healthcare systems and reducing unnecessary expenses. Having access to a robust quality dashboard is helpful. Establish feedback platform for the ground staffs and feedback from other departments, other physicians, patients, and families should be obtained. |
|  | |
| **Resource optimization** | |
| Infrastructure fundamentals - implementing evidence-based care, information technology best practices, efficient care delivery, and the sustainable use of resources | |
|  | 26.Dynamic staff roster to accommodate even distribution according to workload  **Round 1** - A more rationale staffing approach introduced with more even distribution of staff throughout 24-hour period. However, may not be popular with staff and may contribute longer term to retention problems  **Round 2** - Staffing does need to reflect patient workload, but must also consider work-life balance and staff retention. Essential to avoid staff wearing out and exhaustion (very common occurrence during the COVID pandemic). |
|  | 27.Cost-effective sterilization practices  **Round 1** - Ethelene oxide sterilization practice to sterilize and re-use equipment with emphasis on the safety frameworks can effectively reduce the burden of high cost with single use equipment  **Round 2** - There will be regional variations, however, cost-effective sterilization is better than inefficient or no sterilization. Quality can’t be compromised, e.g., distant, but cheaper sterilisation may impair availability of sterile devices when required. |
|  | 28.ICU equipment and devices related  **Round 1** - The choice and purchase of ICU equipment and devices must be supported by evidence-based interventions and ICU staff must be trained.  **Round 2** - Marketing of devices without evidence of patient benefit is common and can lead to expenditure on devices which are not then used, or may even cause harm. |
| Foundational elements – Developing a culture of continuous improvement, through quality improvement methodology | |
|  | 29.Audit of consumable usage  **Round 2** - Consumable usage is one of the crucial drivers of ICU cost and needs to be rationalised |
|  | 30.Reduce the frequency of laboratory tests, radiological tests through QI  **Round 1** - Quality improvement projects aimed at cost-cutting and eliminating waste in terms of overdiagnosis and overtreatment.  **Round 2** - Standards for when tests are ordered can be established, and opportunities may be identified to reduce frequency of testing or apply it in more targeted manners. |
|  | 31.Rationalize the specific indications for transfusion  **Round 1** – ICUs should develop standardized transfusion practice and implement through education or stewardship program.  **Round 2** - There is a large amount of evidence on transfusion targets in critically ill patients and this should be synthesised into guidelines to limit the over-use of transfusion. Triggers for blood products (platelets, fresh-frozen plasma etc..) are less well defined but will also benefit from consensus guidelines where none currently exist. There can be a follow up medical audit where the transfusion practice may be reviewed periodically. |
| Reliability and feedback - embedding safeguards, anti-microbial stewardship and being transparent within the health service | |
|  | 32.Rationalizing the use and having a stewardship approach towards critical resources  **Round 1** - Rationalising the use and having a stewardship approach towards resources of interest like expensive medicines including antibiotics, fluid, steroids, oxygen, triage of ICU beds, requirement of emergency/senior physician (MD) to assess and intervene.  **Round 2** - Daily review of every therapy, asking "is this required, is this beneficial, is this harmful" should be undertaken, and therapies stopped if either not required or actively harmful. |
|  | |
| **ICU/ HDU Admission & Discharge optimization** | |
| Care delivery priorities – integrated and coordinated care delivery, through team-based approaches and shared decision-making | |
|  | 33.Careful discharge planning  **Round 1** - Timely transfer out of patients who are in ICU with objective discharge criteria as well as prevent early discharge and subsequent readmission – requires team approach to decision making, outreach on wards and/ or and step-down units.  **Round 2 -** Readmissions to ICU are predictors of adverse outcomes and are indicators of quality of care, Can consider discharge to home for very stable patients as well. |
|  | 34.Appropriate use of ICU/ HDU resources  **Round 1** - Admission criteria should be strictly followed and be supported by both administration and government bodies to avoid un-necessary admissions (based on VIP or similar recommendations). Additional consideration be given to auditing appropriateness of ICU care (if appropriate patients are being admitted to ICU, either too sick to benefit, too well to benefit)  **Round 2** - ICU resources are scarce and need to be utilized where they are needed most |
| Reliability and feedback - embedding safeguards, anti-microbial stewardship and being transparent within the health service | |
|  | 35.Review of failed discharges/ ICU re-admission to identify opportunities for improvement  **Round 1** - ICU re-admission should be used as a quality benchmark.  **Round 2** - This is important, but needs to be balanced by having a tolerance for readmissions. If no-one is ever readmitted the unit will be holding on to patients too long, and the key question is whether a readmission was preventable. |
|  | 36.Audit of time taken for ICU admission from ED/ Wards  **Round 1** - An important factor in patient outcomes is speed of admission from time of deterioration – among patients admitted with high degree of organ failure, time from decision to admit to actual admission and rates of unanticipated deterioration/ cardiac arrest/ peri-arrest on the wards can all be useful indicators of how rapidly patients are moved to ICU. This should also be linked to ICU capacity versus ICU bed occupancy to see if a hospital has sufficient number of beds.  **Round 2** - Standards should be set for minimising delays. |
| **Expanding the scope of ICU beyond the four walls of ICU** | |
| Infrastructure fundamentals - implementing evidence-based care, information technology best practices, efficient care delivery, and the sustainable use of resources | |
|  | 37.Developing step-down units and long-term care units  **Round 1** - Step down unit (e.g. NIV wards in hospital) and long-term acute care beds (tracheostomy units, rehabilitation units) may allow early discharge of ICU patients (medical or Surgical) as the level of delivered care and staffing is different from ICU. Intensivists should have roles and even be in charge of these beds.  **Round 2** - Long-term facilities are beneficial if the infrastructure exists to support them, e.g., Long-term weaning (or heading towards domiciliary ventilation). However, such units may need to be organised on a regional basis as numbers of patients in each locality are usually small.  One size does not fit all; should be contextualised; emphasize quality, safety, training, competence. |
|  | 38.Appropriate remote monitoring in step-down units  **Round 1** - Remote monitoring systems in step down units, in view of lesser bed side staff, can promptly alert medical and nursing staff to attend deteriorations in high-risk patient, with early interventions.  **Round 2** - Remote monitoring, even if appropriate, could be costly. The utility is dependent on unit organisation, units which are satellites of main ICUs can be covered by ICU staff at lower staff:patient ratios whilst stand-alone units do need monitoring - will depend on local circumstances. |
|  | 39.Utilize tele-ICU to bring down ICU costs as well as support under-served areas  **Round 1** - Tele-ICU support from expert intensivist (each tertiary care center with 2-3 peripheral centres connected for tele consult and provision of reverse referral)  **Round 2** - Whilst it is certainly preferable that all critically ill patients have access to physical presence of a full trained ICU team, in situations where this is not possible telemedicine and remote review can be of assistance Critical to this is ensuring that the advice is based on a full understanding of the patient's situation with access to lab, radiology and clinical data and visual assessment of the patient. It also requires a development of a professional relationship between the remote expert and the on-site team with the ability to provide on-site visits or patient transport if required. May be beneficial in big countries with many remote areas where proper advanced services can't be provided, but it needs proper set up to facilitate critical decision making. |
| Care delivery priorities – integrated and coordinated care delivery, through team-based approaches and shared decision-making | |
|  | 40.Outreach services on wards  **Round 1** - To support ward teams in looking after sicker patients and potentially avoid HDU/ICU admissions, follow up of ICU discharged patients to wards, may help to prevent re-admission and may include follow-up of tracheostomized patients on wards post-ICU discharge  **Round 2** - Limiting factor remains the number of qualified, trained ICU staff to cover the service. |
|  | 41.Introduction of multi-disciplinary rapid response team led by ICU  **Round 1** - Rapid Response Team- (RRT) introduction within the hospital where in a multi-disciplinary team attends to a deteriorating patient, triggered by set clinically monitored parameters, helps in treating them at the earliest opportunity. This may either avoid ICU admission with earlier interventions/ End-of-life planning or facilitate early ICU admission with opportunity for aggressive resuscitation and prevention of organ failure Prevention of cardiac arrest will be a huge impact. This will improve patient safety and undue deterioration elsewhere within the hospital setting. Implementation of early warning scores for hospitalized patients, patients in the emergency room and more standardization in this topic is needed  **Round 2** - Objective evidence of benefit is somewhat scarce - most impact seems to be from setting appropriate treatment limits and ensuring those not suitable for escalation are palliated when they deteriorate, however this is a difficult area to research and observational data plus anecdotal experience do suggest a benefit from ICU outreach with added benefit from medical membership of the team. However, depends on resources, model, staffing. |
|  | |
| **Competencies and Training of staff** | |
| Infrastructure fundamentals - implementing evidence-based care, information technology best practices, efficient care delivery, and the sustainable use of resources | |
|  | 42.Develop and Maintain Competency  **Round 1** - ICUs should be managed by competent intensive care professionals. Additionally, any model of specialized ICUs/ stepdown unit etc, will need fully trained medical and nursing staff.  **Round 2** - Those who work in the unit should be supported in developing and maintaining training in critical care. Skills need to be practiced and reinforced. Competency based training is the key for maintaining quality. |
|  | 43.Continuous training & education  **Round 1** - Continuous training, education as structured training programmes for doctors, nursing staff and nursing assistants, can effectively improve patient care. Basic ICM education could be provided regularly to all nurses and junior doctors in hospitals. Undergraduate medical education should be modified accordingly.  **Round 2** - Developing core curriculums for staff across professional groups, and mapping training onto that curriculum further helps with credentialling and ensuring standardisation of ICU knowledge. The ever evolving knowledge and evidence in critical care mandates a continuous learning process. Essential for the quality of care, needs continuous programs to implement, initially it may look more time, effort, and money consuming but eventually it is better for the quality of care and may reduce cost. |
| Foundational elements – Developing a culture of continuous improvement, through quality improvement methodology | |
|  | 44.Audits of staff competency, training of staffs  **Round 1** - Periodic audits and review with participation of all stakeholders  **Round 2** - Individual KPIs to evaluate ICU physician performance is required but difficult to achieve on individual basis (may be procedure related performance and related complications can be used). OPPE system (Ongoing Personal Performance Evaluation) can be put in place for each physician with subsequent impact on clinical privileges and end of year evaluation. |
|  | |
| **Infection Control measures** | |
| Reliability and feedback - embedding safeguards, anti-microbial stewardship and being transparent within the health service | |
|  | 45.Hand hygiene monitoring  **Round 1** - Hand hygiene monitoring on a continual basis in ICU, will help in reducing hospital acquired infections and cost incurred by the patient. Also, immediate feedback of hand hygiene audit to individuals helps in bolstering the practice to maintain high standards.  **Round 2** - This is simple, and effective. |
|  | 46.Governance of antibiotic stewardship institutionally  **Round 1** - Regular data collection and audit of anti-microbial stewardship, review, and action, may involve simple ways, e.g. "antibiotics delivered to the unit" can allow this without individual patient level data being collected.  **Round 2** - Administrative commitment is crucial for the success of a stewardship program |
|  | 47.Anti-microbial stewardship including Infectious diseases-ICU rounds  **Round 1** - At least one doctor and one nurse should be the reference points regarding the anti-microbial stewardship, raising awareness among colleagues and monitoring the correctness of antibiotic prescription, dilution, and administration, Microbiological surveillance and rational antibiotic use/ controlled by local pharmacy/pharmacologist/ clinical pharmacologists to guide therapy including timely de-escalation of antibiotics will prevent  **Round 2** - Daily rounds with a microbiologist or infection specialist, combined ideally with an antimicrobial/ICU pharmacist can help ensure optimal antibiotic/antifungal use whilst where possible limiting spectrum and duration. However, this could tricky when there is disagreement. |
|  | 48.Healthcare acquired infections prevention  **Round 1** - Spend more time and effort and expense on Healthcare acquired infections (HAI) - the data should be collected and shared with stakeholders on a regular basis. HAI rates be used as a KPI with international/ regional/ local benchmarking.  **Round 2** - Care must be taken to avoid problems with diagnosis and limit the incentives for 'gaming' the system. |
| **Electronic Health Records** | |
| Infrastructure fundamentals - implementing evidence-based care, information technology best practices, efficient care delivery, and the sustainable use of resources | |
|  | 49.Electronic Health Records (EHR) in ICUs  **Round 1** - EHR implementation can reduce the clerical work of medical and paramedical staff and subsequently, increase time available for clinical care. Additionally, large amount of physiological data that is being recorded, may be used for review of critical incidents as well for improving patient safety via clinical governance activities, and development and evaluation of warning systems. However, technology and infrastructures is a big investment in terms of money. Technology can become obsolete in the long term & Little evidence on its cost effectiveness.  **Round 2** - Although this is the way forward, cost-effectiveness is not certain. There is risk of increased documentation burden and also inaccurate documentation |
| Reliability and feedback - embedding safeguards, anti-microbial stewardship and being transparent within the health service | |
|  | 50.Use of electronic health records to increase accountability  **Round 1** - Electronic trails are captured with EHR, which improves accountability of health care professional. However, electronic medical software is costly.  **Round 2** - It is important that staff understand this and that it is not used as a method of criticism or surveillance of staff working practices without consent |
|  | |

Remaining ten interventions that were not moderately or strongly endorsed and/ or had > 15% disagreement.

| Infrastructure fundamentals - implementing evidence-based care, information technology best practices, efficient care delivery, and the sustainable use of resources |
| --- |
| **1.Integrated ICU model preferable to ED-based ICU/ Specialty-based ICUs – 75% yes but…. 15% disagree.**  **Round 1** - Integrated model could be more cost-effective compared to dividing and splitting ICUs. So, department based (such as emergency medicine, internal medicine, Anesthesiology, surgery, pulmonology, cardiac, etc.) ICUs could be centrally managed within the institution. This might lead to rational use of health-care staff and equipment, etc. Department based models have become an example for some countries and this influences ICM organization and training adversely. These will only delay admission to ICU/HDU and the patients that are admitted are likely to be sicker and more complicated. Unfortunately, these will only drive costs up and not down.  **Round 2** -The evidence for either model is not compelling. A plethora of individual units, often run by single organ specialists rather than fully trained intensivists can lead to duplication of costs and fragmentation of care. On the other hand, certain patient groups demand very stable, experienced and trained staff in specific competencies and to optimize patient care, input from various specialities are essential.  Overall, the organization will differ depending on whether it is a high volume / low volume center and also based on individual centers of excellence. Next, there are difference between countries in critical care training. If one does 5 years of critical care training, it might be that an integrated ICU gives equivalent care. If one does only 1-2 years after primary training in another specialty (surgery, anesthesia, pulmonary, neuro, etc) it is highly likely that specialty care is superior to working in an ICU where someone only had a month or two of training in fellowship. |
| **2.A combined ICU & HDU model**  **Round 1** - This would allow easier transition of ICU patients, lesser handover, lesser misses, better quality of care.  **Round 2** - Although a combined model is more convenient, it may cost more to equip/staff every bed like an ICU bed. Flexible ICU/HDU units allow improved access to interventions if a HDU patient deteriorates. If it is a smaller hospital with few inpatients, this model might be cost-effective. High volume ICUs should strongly consider a physically separate HDU with individualized SOPs for resource utilization. |
| **3.Surgical intermediate care unit as cost saving alternatives to ICU care**  **Round 1** - Early interventions in the Surgical intermediate care units may be cost-effective by halting progression of disease.  **Round 2** - An intermediate care unit may be cost saving, for patients on specific pathways, especially those who really need enhanced monitoring for a defined period of time (e.g. post-operative patients after carotid surgery who need BP monitoring, patients with thoracic epidurals following major abdominal/thoraco-abdominal procedures). Such units do not need intensivist staffing; but do need ready access to intensivists for review of patients who deviate from expected trajectories). |
| **4.Creating Critical Care Nurse Consultants, Physician Assistants as part of critical care team**  **Round 1** – Consider developing pathways (Training, governance) with different levels of autonomy to non-physician health care professionals, from basic to advance in different areas of clinical work and setting up procedural teams to provide procedural assistance as a part of multi-disciplinary team  **Round 2** – It may be important for professionals outside of medicine to have opportunities for career development and enhancement, both to aid with retention of their skills and expertise and also to ensure it is recognised. However, the roles of these non-medical consultants do need to be clearly defined, and care must be taken to ensure they are not used in an attempt to provide medical care 'on the cheap', but rather to develop and enhance skills within their own professional sphere of practice. Needs very careful consideration of statuatory compliances, impact on care, training and evaluation avenues, competency of these class of staff. Nurses need more education and autonomy in critically ill patient care, even if not within the critical care nursing consultants pathway |
| **5.Hand-held imaging devices like ultrasound probes attached to smart-phones.**  **Round 1** - Hand-held imaging devices like ultrasound probes attached to smart-phones is becoming very practical and available with a very good and rapid learning curve; apart from usual indications, can be used for difficult IV cannulation.  **Round 2** - Ultrasound is an increasingly used tool in ICU. Portable device may not add anything more than conventional ICU USGs which are already portable enough and have good screen size and resolution. However, in a resource strained setting, such solution may be the only cost-effective way. |
| **6.Low-cost wearable devices to replace the expensive commercial equipment for physiological monitoring**  **Round 2** - The accuracy of low-cost devices may also be lower and hence reduced clinical effectiveness. It is important that they meet appropriate standards of fidelity and reliability when being used for healthcare purposes as opposed to leisure/informal use. |
| **7.Opportunities to use artificial intelligence (AI)**  **Round 1** - May help in development and evaluation of warning systems as well as might aid the physician in deciding which are the most cost-effective tests and interventions in a particular situation  **Round 2** - At present, the evidence for automated alerts, clinical decision support and similar AI-driven approaches is limited, and it is important to assess both their risks and benefits and be careful of alert fatigue and opaque algorithms. AI may be the future and the key for personalised care. |
| **8.Use of disposable items over reusable**  **Round 1** - There is some evidence that disposable items (despite the apparent initial cost) are more cost effective. This enables to save cost from sending to central sterilization, to prevent cross infection, staff safety from exposure to infectious items used by infectious or pandemic patients.  **Round 2** - However, this is still an ambiguous area and needs to be evaluated in light of local laws and manufacturers recommendation, and whether sterilization can be done with adequate safety and monitoring. Reusable may be a good idea in resource limited countries. |
|  |
| Care delivery priorities – integrated and coordinated care delivery, through team-based approaches and shared decision-making |
| **9.Post-intensive care outpatient clinics under the supervision of intensivists**  **Round 2** - The evidence for these clinics is not strong, however it is clear that a large number of patients have persisting illness after ICU admission and these clinics can be of benefit in identifying treatable conditions as well as giving reassurance about recovery |
|  |
| Reliability and feedback - embedding safeguards, anti-microbial stewardship and being transparent within the health service |
| **10.Linking KPIs to physician/ unit renumeration**  **Round 1** - Providing providers transparent access to metrics that identify variations in practice, utilization rates, and performance against internal and external benchmarks * Individual KPIs to evaluate ICU physician performance is required but difficult to achieve on individual basis (may be procedure related performance and related complications can be used).  **Round 2** - Whilst superficially attractive this can lead to gaming - e.g. people simply stop diagnosing VAP, there are also risks of inverse-care, where the sickest and most-vulnerable patients are denied care because they 'make the unit look bad" when they die or have adverse outcomes. Collaborative approaches which seek to benchmark units and find ways to improve are better than punitive ones which seeks to removing funding from units which are struggling. Additionally, KPI are not solely under the control of physicians, the outcomes are frequently determined by a care process. |
